# Prognostic Stratification of Bladder Cancer Patients with a MicroRNA-Based Approach

**DOI:** 10.3390/cancers12113133

**Published:** 2020-10-26

**Authors:** Ilaria Cavallari, Angela Grassi, Paola Del Bianco, Alberto Aceti, Carlotta Zaborra, Evgeniya Sharova, Irene Bertazzolo, Donna M. D’Agostino, Massimo Iafrate, Vincenzo Ciminale

**Affiliations:** 1 Veneto Institute of Oncology IOV—IRCCS, 35128 Padua, Italy; ilaria.cavallari@unipd.it (I.C.); angela.grassi@unipd.it (A.G.); paola.delbianco@iov.veneto.it (P.D.B.); evgeniya.sharova@iov.veneto.it (E.S.); irene.bertazzolo.1@studenti.unipd.it (I.B.); 2 Department of Surgery, Oncology and Gastroenterology, University of Padua, 35128 Padua, Italy; alberto.aceti.1@studenti.unipd.it (A.A.); carlotta.zaborra@studenti.unipd.it (C.Z.); 3 Department of Biomedical Sciences, University of Padua, 35131 Padua, Italy; dm.dagostino@unipd.it

**Keywords:** microRNAs, liquid biopsy, bladder cancer, prognostic stratification

## Abstract

**Simple Summary:**

The majority of patients with bladder cancer are diagnosed before the malignant cells invade the bladder’s muscle wall, and can be treated with surgery. These patients are nevertheless at high risk of disease recurrence and progression to muscle-invasive disease, and must undergo periodic cystoscopy and urine cytology, procedures that are burdensome for the patient and for the healthcare system. We set out to identify a follow-up/risk assessment test that analyzes the levels of a specific class of RNA molecules (microRNAs) in urine samples. Results led to the discovery of a panel of microRNAs in urine samples that identifies high-risk bladder cancer patients with high accuracy and predicts event-free survival. This urine microRNA assay thus holds promise as a noninvasive alternative to current methods for bladder cancer follow-up.

**Abstract:**

Robust non-invasive tests for prognostic stratification of bladder cancer (BCa) patients are in high demand. Following a comprehensive analysis of studies on BCa, we selected a panel of 29 microRNAs (miRNAs) and analyzed their levels in urine and plasma samples in a prospective cohort of 63 BCa patients (32 at high risk of recurrence and 31 low-risk cases) and 37 healthy controls using RT-qPCR. To design an assay suitable for large-scale testing, we applied a hierarchical pipeline to select the miRNAs that were not affected by confounding factors such as haematuria and urine specific gravity, and exceeded stringent cut-off criteria (fold change > 2.5 and *p*-value < 0.005). Using a two-step decision tree based on the urine levels of miR-34a-5p, miR-200a-3p and miR-193a-5p, normalized against miR-125b-5p, patients could be classified as high- or low-risk with a sensitivity of 0.844, specificity of 0.806 and accuracy of 0.825. Furthermore, univariate Cox proportional hazards regression analyses indicated that increased urine levels of miR-29a-3p, miR-34a-5p, miR-193a-5p, miR-200c-3p, miR-205-5p and miR-532-5p were associated with a shorter event-free survival (hazard ratios > 3.1, *p*-value < 0.05). Taken together, our findings suggest that measuring the urine levels of these miRNAs could provide a novel cost-effective, noninvasive test for risk assessment of BCa patients.

## 1. Introduction

Worldwide, bladder cancer (BCa) is the fourth most frequently diagnosed malignancy in men and the 15th in women [1]. BCa is classified as either non-muscle invasive (NMIBC) or muscle-invasive (MIBC). NMIBC represents the most common form (70–80%) and is primarily treated by transurethral resection. While haematuria is the most common symptom at presentation in patients with advanced disease, many patients in earlier stages with micro-haematuria are not adequately diagnosed. NMIBC displays a high recurrence rate (50–70%) and patients require strict follow-up by cystoscopy (every 3–6 months for 5 years) and urine cytology, which imparts considerable stress to the patient and costs for the health care system [2,3]. Optimization of treatment requires patient stratification into low and high-risk groups based on pathological grading and staging. Accurate non-invasive tests that allow a better stratification of BCa are thus in high demand. Recent studies showed that aberrantly expressed microRNAs (miRNAs) are released by cancer cells into body fluids. In the case of BCa, urine is likely to represent an ideal diagnostic biofluid, as it is in direct contact with cancer cells. Many studies have investigated cell-free circulating miRNAs (cfmiRNAs) in the plasma and urine of BCa patients. However, so far, a consensus signature has not emerged, possibly due to differences in preanalytical procedures, data normalization and confounding factors, which, in the case of urine samples, include haematuria and differences in urine concentration [4,5]. The present study led to the identification of a panel of urine cfmiRNAs capable of distinguishing high-risk from low-risk BCa patients and to a pilot investigation of their prognostic role.

## 2. Results

### 2.1. Selection Pipeline to Identify Relevant miRNAs

Quantitative real-time Reverse transcription-polymerase chain reaction (qRT-PCR) was used to measure the urine and plasma levels of cfmiRNAs in a prospective cohort of 63 BCa patients and 37 control individuals (Table 1 and Tables S1–S3). A total of 32 patients were at high risk of recurrence, 27 were low-risk; 4 with intermediate risk were grouped together with low-risk patients. Urine and plasma samples were centrifuged and miRNA analyses were carried out on the cell-free supernatant. 

**Figure 1:**
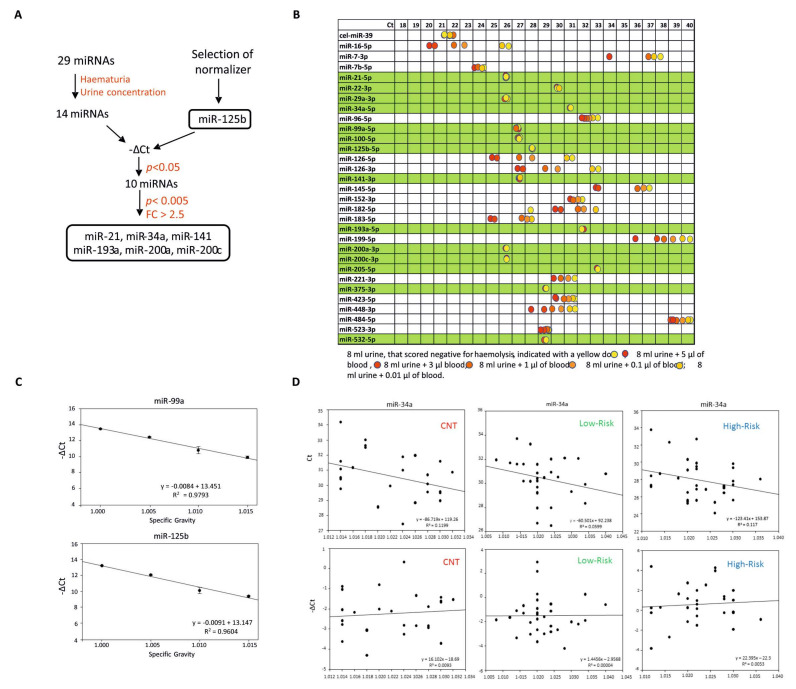
Study design. (**A**). miRNA selection pipeline. Starting from a total of 29 miRNAs, we assessed the common confounding factors haematuria and urine specific gravity (SG) to verify the robustness of miRNA quantification. Selective criteria were applied to reduce the number of miRNAs to be tested. (**B**). Effects of haematuria on urine miRNA levels. The indicated volumes of haemolyzed blood were added to a urine sample from a control volunteer, to construct a 6-point “curve”. miRNAs listed in the table were analyzed. Cel-miR-39 was added to the healthy control sample to verify the quality of the extraction. The levels of miRNAs highlighted in green were not influenced by the presence of blood. (**C**). Selection of normalizer. A control urine sample was diluted with water to obtain different SG. The graphs show the linear regression of −ΔCt ($\Delta Ct_{miR}=Ct_{miR}-Ct_{cel-miR-39}$) of miR-99a and miR-125b vs. urine SG. The assay was performed in triplicate. Both normalizers correlated with urine SG, as indicated by their coefficient of determination *R^2^* value (see plots). (**D**). Correlation with urine SG. The graphs report linear regression of miR-34a Ct or −ΔCt vs. urine samples’ SG in controls, low-risk and high-risk patients (values of coefficient of determination *R^2^* are indicated). The graphs show that miR-34a levels did not correlate with SG when the miRNA was normalized for miR-125b (−ΔCt) (bottom panels, see *R^2^* values).

To guide a rational selection of relevant cfmiRNAs, we designed a selection pipeline (Figure 1A) following a comprehensive analysis of studies on BCa, and identified a panel of 29 miRNAs (miR-7-3p [6,7], miR-7b-5p [7], miR-21-5p [8,9], miR-22-3p, miR-29a-3p [7], miR-34a-5p [8], miR-96-5p [10], miR-99-5p, miR-100-5p [11], miR-125b-5p [12], miR-126-5p [7], miR-126-3p [13], miR-141-3p [14,15,16], miR-145-5p [8], miR-152-3p [13], miR-182-5p [10,13], miR-183-5p [10], miR-193-5p [6], miR-199-5p [13], miR-200a-3p, miR-200c-3p [8,14,15,16], miR-205-5p, miR-221-3p [8], miR-375-3p, miR-423-5p, miR-523-3p [8], miR-448-3p [6], miR-484-5p [17] and miR-532-5p [7]). As several miRNAs are abundant in red blood cells (RBC) and haematuria is a very common sign of bladder disease (including BCa), we first tested the urine levels of the selected cfmiRNAs following the addition of increasing volumes of blood to a control urine sample from a healthy individual. We measured miR-16, as it known to be abundant in RBC [18] and spike-in cel-miR-39 that was added to the sample to control the preanalytical steps. Results showed that the levels of miR-21, miR-22, miR-29a, miR-34a, miR-99a, miR-100, miR-125b, miR-141, miR-193a, miR-200a, miR-200c, miR-205, miR-375 and miR-532 were not affected by the presence of blood (Figure 1B). Based on these findings, these 14 cfmiRNAs were selected for further analysis. An unbiased analysis method was used for the selection of miRNAs for data normalization; miR-125b and miR-99a showed the lowest standard deviation in all the samples examined (Table S4, Figure S1). To normalize the cfmiRNA measurements for differences in urine concentration, a control urine sample was diluted to obtain different specific gravity (SG) values and the correlation between cfmiRNA levels and SG was evaluated (Figure 1C). Cel-miR-39 was used as exogenous spike-in control and relative expression of internal miRNA normalizers was calculated as $\Delta Ct_{miR}=Ct_{miR}-Ct_{cel-miR-39}$. The −ΔCt of both miR-125b and miR-99a correlated with SG; miR-125b was selected because of its lower standard deviation. Normalizing the cfmiRNA values against miR-125b produced measurements that were independent of urine concentration (shown for miR-34a in Figure 1D; see also Figure S2).

### 2.2. Identification of a Urine cfmiRNA Panel to Discriminate High-Risk from Low-Risk BCa Patients

qRT-PCR analyses indicated that the urine levels of miR-21, miR-22, miR-29a, miR-34a, miR-141, miR-193a, miR-200a, miR-200c, miR-205 and miR-532 normalized for miR-125b were significantly higher (*p* < 0.05) in high-risk patients compared to low-risk patients (Figure S3A and Table S5). Similar results were obtained by normalizing miRNAs for miR-99a (Figure S3B). No significant difference was observed comparing low-risk patients with healthy controls (Figure S3A,B).

**Figure 2:**
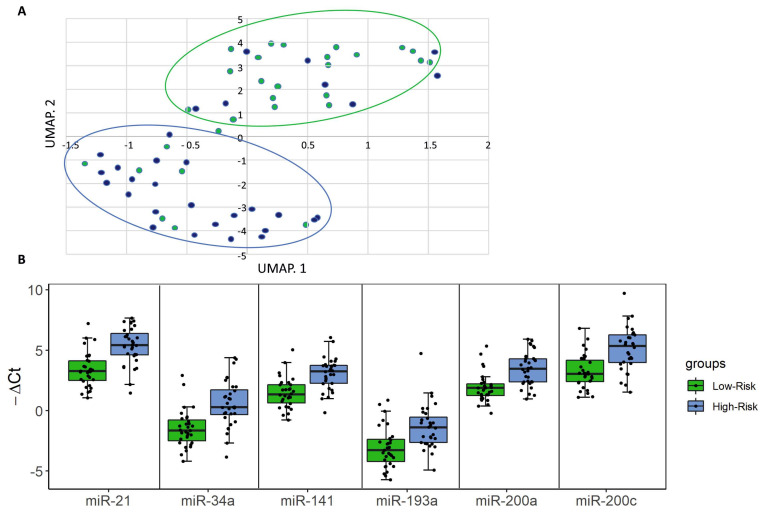
A urine cfmiRNA panel to discriminate high-risk from low-risk bladder cancer (BCa) patients. (**A**) Uniform Manifold Approximation and Projection (UMAP) analysis. Using a first cut-off of *p* < 0.05, we identified 10 miRNAs (miR-21, miR-22, miR-29a, miR-34a, miR-141, miR-193a, miR-200a, miR-200c, miR-205 and miR-532) and subjected them to UMAP analysis using $-\Delta Ct_{x}$ values. The UMAP plot shows that most of the high-risk patients (blue dots) and low-risk patients (green dots) segregated into distinct clusters. (**B**) Box plot analysis of 6 cfmiRNAs upregulated in high-risk BCa patients. With more stringent selection criteria of fold change (FC) > 2.5 and *p* < 0.005, we further restricted the analysis to a 6-cfmiRNA panel (miR-21, miR-34a, miR-141, miR-193a, miR-200a and miR-200c). The graphs show the −ΔCt distribution of these cfmiRNAs in low-risk (green) and high-risk (blue) patients. Dots represent individual patients. FCs and *p* value are indicated in Table S5.

Multidimensional Uniform Manifold Approximation and Projection (UMAP) analysis of these urine cfmiRNAs yielded two distinct clusters that distinguished the majority of the high-risk patients from low-risk patients (Figure 2A, blue and green dots, respectively). To evaluate the predictive power of these markers, a univariate logistic regression model was developed and the areas under receiver operating characteristic (ROC) curves were calculated (Table S5).

We next applied more stringent selection criteria in the comparison of high-risk vs. low-risk patients, i.e., fold change (FC) > 2.5 and Bonferroni-adjusted *p*-value < 0.005 (Figure S4). miR-21, miR-34a, miR-141, miR-193a, miR-200a and miR-200c fulfilled these criteria (Figure 2B), and exhibited area under the ROC curve (AUC) values > 0.75 (Table S5). Details of the univariate logistic regression models for the six selected miRNAs, with odds ratios, 95% confidence intervals and *p*-values are shown in Table S6.

As cigarette smoking and gender are major risk factors for BCa, we compared the urine levels of these miRNAs in non-smokers (Non-S) vs. smokers (S) and in female (F) vs. male (M) BCa patients. Results showed that patients within the same risk group of BCa exhibited cfmiRNA levels that were not significantly different in smoking vs. non-smokingor male vs. female patients (Figure S5).

To further evaluate our results, we carried out an unsupervised analysis of all the ratios between the expression values of all 14 cfmiRNAs, as previously described [19,20]. 91 miRNA ratios were generated (Table S7). Notably, the results of this unsupervised approach confirmed the panel of six cfmiRNAs (miR-21, miR-34a, miR-141, miR-193a, miR-200a, miR-200c) as the most significant indicators of high-risk of recurrence using miR-125b as normalizer.

An evaluation of miR-21, miR-29a, miR-34a, miR-99a, miR-100, miR-125b, miR-141, miR-193a, miR-200a and miR-200c in plasma from high- and low-risk BCa patients and controls revealed that their levels were very low and not significantly different among the three groups of individuals (Figure S6 and Table S8), suggesting that these miRNAs are likely to be shed into the bladder by cancer cells rather than originating from transrenal filtration of plasma miRNAs.

### 2.3. Urine Levels of miR-34a, miR-200a and miR-193a Stratify High- and Low- Risk BCa Patients

**Figure 3:**
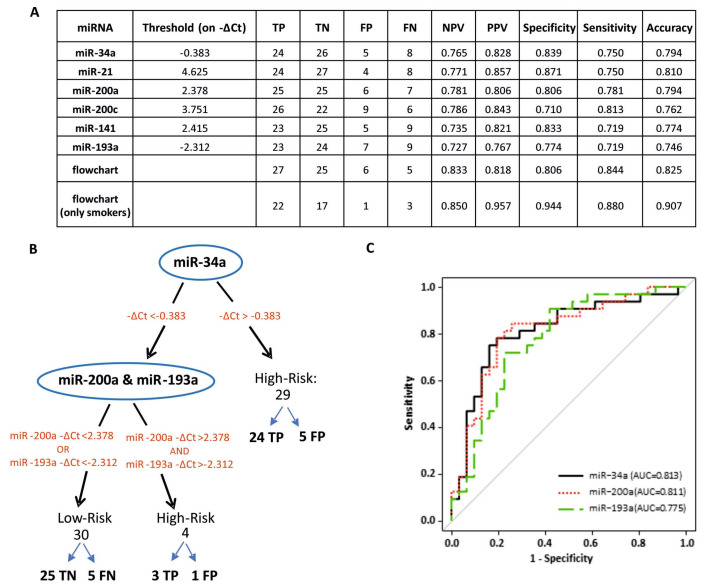
Urine cell-free miR-34a, miR-193a and miR-200a for BCa risk stratification. (**A**). Assessment of the performance of different patient stratification strategies. The table shows the thresholds and metrics for the evaluation of each biomarker’s performance in our data set. The miRNAs and the flowchart were evaluated for their ability to classify patients as high-risk or low-risk. Abbreviations: TP = true positive; TN = true negative; FP = false positive; FN = false negative; NPV = negative predictive value; PPV = positive predictive value. (**B**) Two-step decision flowchart for identification of high-risk BCa. A flowchart with miR-34, miR-193a and miR-200a as biomarkers and miR-125b as normalizer was designed to minimize the number of false negatives and improve the performance of individual miRNAs. The patients with − ∆Ct_miR-34a_ > −0.383 are classified as high-risk. The remaining patients pass to the second step where they are evaluated for miR-200a and miR-193a; if the patient shows both −∆Ct_miR-200a_ > 2.378 and −∆Ct_miR-193a_ > −2.312, he/she is classified as high-risk, if the patients show −∆Ct_miR-200a_ < 2.378 and/or −∆Ct_miR-193a_ < −2.312 he/she is classified as low-risk. (**C**) Receiver operating characteristic (ROC) curve analysis. Shown are ROC curves and corresponding area under the curve (AUC) values for the cfmiRNAs employed in the flowchart.

The Youden index was used to determine the best threshold to discriminate between low- and high-risk BCa patients for each selected miRNA, and accuracy, sensitivity, specificity, negative predictive value (NPV) and positive predictive value (PPV) were calculated (Figure 3A). 

To further streamline our assay for potential application to large scale testing, starting from the −ΔCt thresholds already determined for individual miRNAs, we designed a data-driven flowchart to minimize the number of false negatives using a parsimony principle (Figure 3B). Using this approach, we defined a two-step decision tree based on the levels of miR-34a, miR-193a and miR-200a as biomarkers of high-risk BCa with miR-125b as normalizer. Figure 3C shows the ROC curves and the corresponding AUC for these cfmiRNAs. 

The proposed flowchart classified the high- and low-risk patients with a sensitivity of 0.844, specificity of 0.806 and accuracy of 0.825, with six false positives and five false negatives. When applied only to patients identified as smokers, this flowchart yielded a sensitivity of 0.880, a specificity of 0.944 and an accuracy of 0.907, with only one false positive and three false negatives (Figure 3A), suggesting that the assay is more accurate for patients with smoking as a risk factor. 

### 2.4. Association between Urine cfmiRNAs and Event-Free Survival of BCa Patients

**Figure 4:**
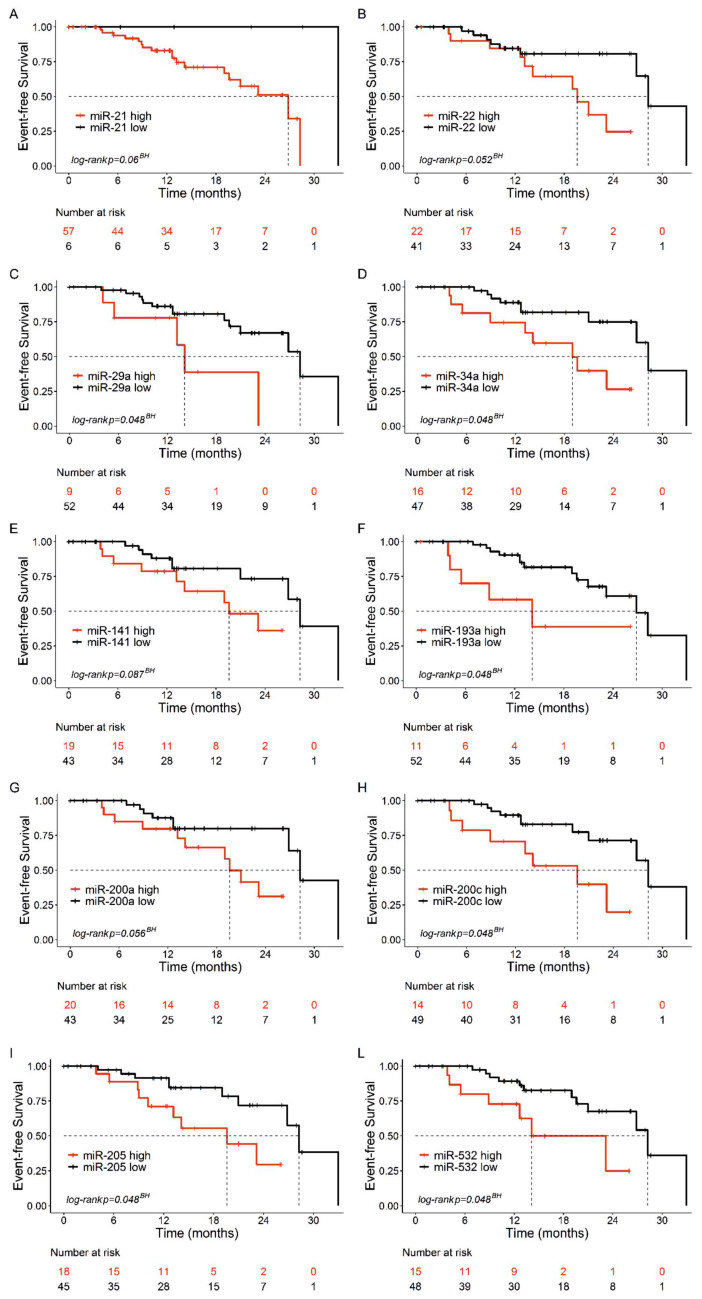
Kaplan–Meier curves of event-free survival (EFS) in bladder cancer patients with high/low urine levels of the indicated miRNAs. (**A**) miR-21: Not Estimable. (**B**) miR-22, hazard ratio (HR) = 3.0, confidence interval (CI):1.1–8.3. (**C**) miR-29a, HR = 3.5, CI: 1.2–10.2. (**D**) miR-34a, HR = 3.3, CI: 1.2–8.9. (**E)** miR-141, HR = 2.5, CI: 0.9–6.6. (**F**) miR-193a, HR = 3.8, CI:1.3–11.0. (**G**) miR-200a, HR = 2.9, CI:1.0–8. (**H**). miR-200c, HR = 3.5, CI:1.3–9.3. (**I**) miR-205, HR = 3.1, CI: 1.1–8.2. (**L**) miR-532, HR = 3.2, CI: 1.2–8.6. The EFS was defined as the time from treatment start to the date of documented disease recurrence, progression or death. BH: Benjamini-Hochberg-adjusted *p*-value.

Next, we analyzed the association between the urine levels of these miRNAs and clinical outcome by using univariate Cox proportional hazards regression modelling. Clinical outcome was analyzed in terms of event-free survival (EFS), defined as the time from the start of treatment to the date of a documented disease recurrence, progression or death; patients who did not develop an event during the study period were censored at the date of the last observation (see Materials and Methods for details). As shown in Figure 4, resulting Kaplan–Meier curves indicate that high levels of miR-29a, miR-34a, miR-193a, miR-200c, miR-205, and miR-532 were associated with a shorter event-free survival with hazard ratios (HR) ranging from 3.1 (miR-205) to 3.8 (miR-193a) (Figure 4, Table S9). 

At a median follow-up of 12.9 months, no clinical characteristic was significantly associated with event-free survival (Table S10).

## 3. Discussion

Our findings suggest that measuring the urine levels of miR-34a, miR-193a, and miR-200a, with normalization against miR-125b, could represent a robust, noninvasive test for risk assessment of BCa patients. The miRNAs on this panel are not affected by the potential confounding factors of urine concentration and haemolysis, and miR-125b was identified as a suitable normalizer based on its lower SD and better performance in the statistical test employed in this study.

The accuracy of this 4-miRNA test (0.825) is comparable to that of a 6-miRNA panel (miR-16, miR-21, miR-34a, miR-200c, miR-205, and miR-221) identified by Sapre et al. [8] to detect tumor recurrence in BCa patients (AUC = 0.85). The fact that our intermediate 6-miRNA panel (Figure 3A) and that of Sapre et al. have only three miRNAs in common (miR-21, miR-34a and miR-200c) is in line with the general limited consensus among studies aimed at identification of miRNAs for BCa diagnosis and risk stratification. Studies aimed at identifying miRNAs as diagnostic markers of BCa show substantial differences in pre-analytical approaches and limited overlap of the results [7,10,13,21,22,23]. These discrepancies could in part reflect differences in the preparation of the starting sample (unfractionated urine, sediment or supernatant after centrifugation, extracellular vesicle fraction), miRNA purification methods, the approach to data normalization, and properties of the starting sample, such as urine concentration and presence of haematuria. For example, while we analyzed centrifuged urine supernatants, the study by Sapre et al. employed unfractionated urine samples and normalized miRNA data against urine osmolality [8]. The inclusion of a centrifugation step in the sample preparation protocol is a critical factor, as some miRNAs may be enriched in platelets and/or exosomes. In the present study, we analyzed cell-free urine and plasma supernatants that were centrifuged at low speed and thus likely included extracellular vesicles but not platelets or other cells [24]. 

A study of centrifuged urine supernatants by Wang et al. [25] identified reduced levels of miR-214 (normalized against snRNA RNU6 and snoRNA RNU48) as a potential diagnostic and prognostic marker of BCa (AUC = 0.838). However, the use of small nuclear/nucleolar RNAs such as RNU6, RNU44 and RNU48 as normalizers is controversial, due to their different length, processing, and subcellular compartmentalization compared to miRNAs. Piao et al. [23] recently proposed measuring the miR-6124/miR-4511 ratio in centrifuged urine supernatants to distinguish bladder cancer from benign haematuria. Aside from this study, the problem of haematuria has largely been ignored in the search for urine miRNA biomarkers of BCa. In our hands, some of the miRNAs cited in studies of BCa appeared to be abundant in RBC and thus potentially affected by the presence of haematuria; therefore, our approach was to eliminate these miRNAs from further consideration (Figure 1B).

The detection of high levels of miR-34a and miR-200a in the urine of high-risk BCa patients is intriguing, given that miR-34a was reported to be downregulated in BCa tumor samples [26,27,28], while all five members of the miR-200 family show a higher level of expression in tumors compared to normal epithelium, but decrease in cells from high-grade BCa [14].

miR-34a expression is lost or downregulated in a wide variety of cancers, and is generally considered to act as a tumor suppressor miRNA by influencing the expression of a plethora of genes involved in cell proliferation, autophagy, metabolism, apoptosis, senescence, stemness, epithelial-to-mesenchymal transition (EMT) and motility [29,30]. miR-34a targets identified in the context of bladder cancer cells include CD44 [27] and TCF1 and LEF1 [31].

The miR-200 family members also play an important role in controlling the EMT, tumor progression and metastasis [32,33,34]. Interestingly, increased levels of circulating miR-200 family members are associated with poor prognosis and are independent predictors of poor clinical outcome in other solid tumors [35,36,37,38,39,40]. An important function of these miRNAs is to suppress the EMT-specific transcription factor ZEB1. In situ studies of colon cancer lesions showed that miR-200 family levels are reduced at the invasion front of primary tumors (the site of EMT) mainly though epigenetic silencing, and are upregulated in the metastatic colonies, in which a mesenchymal–epithelial transition (MET) program is activated [39]. 

miR-193a is involved in many cancer histotypes, including bladder cancer [41]. Interestingly, in lung cancer patients, the levels of cell-free miR-193a-5p were increased in the peripheral blood, while this miRNA was downregulated in cancer cells [42]; this effect was associated with DNA methylation of the miR-193a locus [43]. Functional analyses revealed that re-expression of miR-193a inhibited cell proliferation, colony formation, migration, and invasion in lung cancer cells, and partially reversed the EMT induced by tumor growth factor-β1 (TGF-β1) [43].

Future studies should be addressed at validating the miRNA panel in the context of a large prospective cohort, at assessing whether these circulating miRNAs are free or contained in vesicles, and whether they, in their extracellular form, have a functional role in BCa progression.

## 4. Materials and Methods

### 4.1. Patients

The study was performed in accordance with the declaration of Helsinki and was approved by the local Human Ethics Committee “Comitato Etico per la Sperimentazione Clinica della Provincia di Padova” (4095/AO/2017). Informed consent was obtained from all participants.

In collaboration with the Urology Clinics of the University of Padova, 63 therapy-naïve BCa patients (31 low-/intermediate risk patients and 32 high-risk patients, 11 of whom had MIBC) and 37 controls were enrolled in this study (Table 1 and Tables S1–S3). The risk stratification of the patients was carried out according to the European Association of Urology guidelines [44].

### 4.2. Urine cfmiRNA Purification

A sample of 20 ml freshly voided mid-stream urine was collected from the patients, in the morning before cystoscopy. Samples were immediately supplemented with Norgen’s urine preservative (Norgen Biotek Corp., Thorold, Canada), and processed within 2 hours after collection. Standard sterile disposable polypropylene containers were used. The samples were centrifuged at 1200× *g* for 7 min to obtain cell pellets and cell-free urine and stored −80 °C. Before storage, the samples were checked to test for the presence of protein, glucose, ketones, intact or haemolized RBC, bilirubin, urobilinogen, nitrite and leukocytes, as well as the pH and SG (Siemens Multistix 10 SG, Milan, Italy). The SG of the samples was also measured with a refractometer with automatic temperature compensation. The urine parameters in patients and controls are reported in Table S11. miRNAs were extracted from 2 mL of cell-free urine supernatants with the Urine microRNA Purification Kit (Norgen Biotek Corp.) following the manufacturer’s instructions. miRNAs were eluted in 60 µL of Elution Buffer.

### 4.3. Plasma cfmiRNA Purification

Blood samples were collected in ethylenediamine tetraacetic acid (EDTA)-containing tubes and processed within 2 h. Five millimeters of whole blood were layered over 4 mL of Ficoll-Paque PLUS (GE Healthcare) and centrifuged at 580× *g* for 30 min at room temperature. The upper phase (≤2 mL) was centrifuged at 2500× *g* for 15 min to eliminate platelets and residual cells. Plasma aliquots were stored at −80 °C and used for not more than two freeze–thaw cycles. Total RNA was extracted from 600 µL of plasma with the NucleoSpin miRNA plasma kit (Macherey-Nagel, Düren, Germany) following the manufacturer’s instructions. miRNAs were eluted in 50 µL of Elution Buffer.

### 4.4. Analysis of miRNAs in Urine and Plasma Samples

A 2.5-µL aliquot of the purified miRNA fraction was used for first-strand cDNA synthesis in a 6.5 µL reaction volume, using the TaqMan miRNA Reverse Transcription kit and miRNA-specific stem-loop primers (Thermo Fisher Scientific, Waltham, MA USA ). cDNAs (2.5 µL) were amplified for 45 cycles using TaqMan miRNA primers and probes (Thermo Fisher Scientific) and LightCycler 480 PCR Master Mix (Roche Diagnostics, Basel, Switzerland). No-RT and no-template negative controls were included. The amplification reactions were performed in a LightCycler 480 II thermal cycler (Roche). Signals were quantified using the second derivative maximum method (Software Version 1.5, Roche). The following Taqman miRNA kits (primers and probes, Thermo Fisher Scientific, Waltham, MA, USA) were employed: cel-miR-39 ID:000200, hsa-miR-let-7b-5p ID:002619, hsa-miR-16-5p ID:000391, hsa-miR-21-5p ID:000397, hsa-miR-22-3p ID:000398, hsa-miR-29a-3p ID:002112, hsa-miR-34a-5p ID: 000426, hsa-miR-96-5p ID:000186, hsa-miR-99a-5p ID:000435, hsa-miR-100-5p ID:000437, hsa-miR-125b-5p ID:000449, hsa-miR-126-5p ID:002228, hsa-miR-126-3p ID:000451, hsa-miR-145-5p ID:002278, hsa-miR-152-3p ID:000475, hsa-miR-141-3p ID:000463, hsa-miR-182-5p ID:002334, hsa-miR-183-5p ID:002269, hsa-miR-193a-5p ID:002281, hsa-mir-199a-5p ID:000498, hsa-miR-200a-3p ID:000502, hsa-miR-200c-3p ID:002300, hsa-miR-205-5p ID:000509, hsa-miR-221-3p ID:000524, hsa-miR-375-3p ID:000564, hsa-miR-423-5p ID:002340, hsa-miR-448-3p ID:001029, hsa-miR-484-5p ID:00182, hsa-miR-523-5p ID:002386, hsa-miR-532-5p ID:001518.

### 4.5. Normalization

For each miRNA and for each patient, relative expression was calculated as $\Delta Ct_{miR}=Ct_{miR}-Ct_{\mathrm{normalizer}}$. MiRNA fold change (FC) between two groups of patients $\left(G1,\;G2\right)$ was then calculated as $FC=2^{-\Delta \Delta Ct_{miR}}$, where $\Delta \Delta Ct_{miR}=\bar{\Delta Ct_{miR}\left(patient\_G1\right)}-\;\bar{\Delta Ct_{miR}\left(patient\_G2\right)}.$

### 4.6. Statistical Analysis

UMAP was performed on 10 continuous markers (−ΔCt of 10 miRNAs listed in the legend to Figure 2A) to reduce data dimensionality and identify groups of patients. A two-tailed Wilcoxon rank sum test, followed by the Bonferroni multiple testing correction, was used to identify cfmiRNAs significantly different between the low-risk and high-risk groups. Post-hoc power analysis was performed using a nonparametric resampling technique (10,000 replications). A univariate logistic regression model was built to evaluate the ability of each cfmiRNA on a log_2_-scale to predict high-risk patients. ROC curves were plotted for each miRNA and the area under the ROC curve (AUC) was estimated to compare the most informative cfmiRNAs. Optimal thresholds were determined using the Youden’s J statistic. Sensitivity, specificity, negative predictive value (NPV), positive predictive value (PPV) and accuracy were assessed. Statistical analysis was performed in the R environment using customized code and two pre-built packages (umap and pROC).

Clinical outcome was analyzed in terms of event-free survival (EFS), defined as the time from the start of treatment to the date of a documented disease recurrence, progression or death. Patients who did not develop an event during the study period were censored at the date of the last observation. Survival probabilities were estimated using the Kaplan–Meier method and compared among strata using the log-rank test. We corrected *p*-value for multiple comparisons according to Benjamini–Hochberg correction [45]. The median survival probabilities were reported with their 95% confidence intervals (CI). 

The association of clinical characteristics and the 10 miRNAs with survival was investigated with univariate Cox proportional hazards regression. No deviation from the proportional hazards assumption was found by the test statistic of Grambsch and Therneau [46]. In order to distinguish low- and high-risk patients, the miRNAs were dichotomized with cut-off points corresponding to the most significant relation with the outcome, estimated from maximally selected log-rank statistics for values between the 10% and 90% quantile using the upper boundary of the *p*-value by Hothorn and Lausen [47]. Hazard ratios with their 95% confidence interval are reported in the legend to Figure 4. All statistical tests were two-sided and a *p*-value < 0.05 was considered statistically significant. 

We also cross-validated the cut-off procedure in order to evaluate the robustness of the models. The cut-point estimation was carried out on 10,000 bootstrap samples, the miRNA variables were categorized in order to estimate their association with the time to event, and the *p*-value was calculated for each bootstrap replicate. Although the variability of the cut-point distribution was not negligible, more than 80% of the bootstrap replicates produced significant *p* value similar to those found in the original data set for 9 out of 10 miRNAs. The exception was miR-141, which yielded *p* ≤ 0.05 in 68% of the bootstrap samples and *p* = 0.0642 in the original dataset.

Statistical analyses were performed using RStudio (RStudio: Integrated Development for R. RStudio Inc., Boston, MA, USA).

## 5. Conclusions

By using an evidence-based approach to select candidate miRNAs and controlling for important variables associated with quantification of miRNAs in urine samples, we were able to design a robust, streamlined assay based on a decisional flowchart with high predictive performance. Although this assay needs to be validated with a larger prospective cohort of BCa patients, the encouraging results obtained thus far support the potential for urine miRNA analysis as a noninvasive approach for accurate risk assessment of BCa patients, with reduced cost and burden to the patient compared to current procedures.

## Figures and Tables

**Table 1:** Patients’ characteristics.

Characteristics		N	%
Gender	Female	16	25.4
	Male	47	74.6
Risk	Low	27	42.9
	Intermediate	4	6.3
	High	21	33.3
	MIBC	11	17.5
Smoking	Yes	15	23.8
	No	16	25.4
	Ex	32	50.8
Intracavitary therapy	Yes	25	39.7
	No	38	60.3

Median age of patients: 71 years (Q1 = 66; Q3 = 77). Median follow-up of patients: 12.9 months (Q1 = 8.5; Q3 = 22.3).

## Supplementary Materials

The following are available online at https://www.mdpi.com/2072-6694/12/11/3133/s1. Table S1: Clinico-pathological features of 31 low-/intermediate risk BCa patients examined. Table S2: Clinico-pathological features of 32 high-risk and MIBC patients examined. Table S3: Features of control group. Table S4: Mean Ct and standard deviation (SD) of urine cfmiRNAs analyzed in the total cohort of patients and controls. Table S5: Evaluation of urine cfmiRNAs as biomarkers of low-risk vs high-risk BCa. Table S6: Univariate logistic regression models for the six miRNAs selected as urine biomarkers of high-risk BCa. Table S7: Ratios generated by combinations of the 14 urine cfmiRNAs examined. Table S8: Mean Ct and standard deviation (SD) calculated for miRNAs in plasma samples. Table S9: Univariate analysis of urine miRNAs associated with EFS in bladder cancer patients. Table S10: Univariate analysis of clinical characteristics of bladder cancer patients associated with EFS. Table S11: Urine parameters in patients and controls. Figure S1: Boxplots with the distribution of the two best candidate normalizers in urine. Figure S2: Evaluation of urine specific gravity and miRNA detection. Figure S3: Distribution of urine miRNA levels in controls and BCa patients. Figure S4: Scatter plot showing the criteria used to identify the six most upregulated urine miRNAs in high-risk vs low-risk patients. Figure S5: Evaluation of confounding factors: smoking and gender. Figure S6: Plasma miRNA levels across groups.

## List of Abbreviations

BCa	Bladder Cancer
NMIBC	Non-Muscle Invasive Bladder Cancer
MIBC	Muscle Invasive Bladder Cancer
cfmiRNA	cell-free circulating miRNA
SG	urine Specific Gravity
FC	Fold Change
SD	Standard Deviation
Q	quartile,
RBC	red blood cells
RT-qPCR	Reverse Transcription-quantitative Real Time PCR
UMAP	Uniform Manifold approximation and Projection
