# Supplementary material for: Prognostic Stratification of Bladder Cancer Patients with a MicroRNA-Based Approach
Source: Cancers (Basel). 2020 Oct 26;12(11):3133. doi: 10.3390/cancers12113133 (PMC7692037; doi:10.3390/cancers12113133)
Supplement: Supplementary file 1 [file cancers-12-03133-s001.pdf]

cancers

MDPI

Article

Prognostic Stratification of Bladder Cancer Patients with a MicroRNA-based Approach

Ilaria Cavallari, Angela Grassi, Paola Del Bianco, Alberto Aceti, Carlotta Zaborra, Evgeniya Sharova, Irene Bertazzolo, Donna M. D'Agostino and Massimo Iafrate and Vincenzo Ciminale.

Table S1. Clinico-pathological features of the 31 low-/intermediate risk BCa patients examined.

ID	Gender	Age at Diagnosis	Clinical stage at diagnosis	Risk	Smoker (1 = yes)
#1	F	54	TaLG	low	1
#2	M	71	TaLG	intermediate	1
#4	F	68	TaLG	low	1
#5	M	67	TaLG	low	1
#6	F	82	PUNLMP	low	0
#7	M	75	TaLG	low	1
#8	M	71	TaLG	low	1
#9	F	73	LG	intermediate	1
#10	M	67	TaLG	low	1
#11	F	53	TaLG	low	0
#12	M	71	PUNLMP	low	0
#13	M	63	TaLG	low	1
#14	M	66	TaLG	low	1
#15	F	72	PUNLMP	low	1
#16	M	75	PUNLMP	low	0
#17	F	88	Ta LG	low	0
#18	M	65	Ta LG	low	1
#20	M	66	PUNLMP	low	1
#21	F	70	TaLG	low	0
#22	M	70	PUNLMP	low	1
#23	M	56	PUNLMP	low	0
#24	F	57	PUNLMP	low	0
#25	M	70	TaLG	intermediate	1
#26	M	67	PUNLMP	low	1
#27	M	64	TaLG	low	1
#28	F	85	PUNLMP	intermediate	0
#29	F	83	TaLG	low	0
#30	M	84	TaLG	low	1
#31	M	66	TaLG	low	0
#32	F	79	TaLG	low	1
#33	M	83	PUNLMP	low	1

TaLG = stage Ta low-grade, PUNLMP = papillary urothelial neoplasm of low malignant potential. Smoker 0 = No history of cigarette smoking; Smoker 1 = currently smoking or previous history of cigarette smoking.

Table S2. Clinico-pathological features of the 32 high-risk and MIBC patients examined.

ID	Gender	Age at Diagnosis	Clinical stage at diagnosis	Risk	Smoker (1 = yes)
#3	M	74	Ta LG	high	1
#19	M	74	Ta LG	high	1
# 34	F	58	T2 + Cis	MIBC	1
#35	F	59	T2 + Cis	MIBC	1
#36	M	78	Cis	high	1
#37	M	66	TaHG	high	1
#38	F	88	T1HG	high	1
#39	M	81	T1HG	high	0
#40	M	69	T2	MIBC	1
#41	F	69	T1HG	high	1
#42	M	82	mT1HG	MIBC	1
#43	M	62	TxHG	high	1
#44	M	75	T2	MIBC	1
#45	M	71	T1HG+ Cis	high	1
#46	M	76	TaHG + Cis	high	1
#47	M	83	T2	MIBC	1
#48	M	67	T2	MIBC	1
#49	M	73	T1HG	high	0
#50	M	80	T2	MIBC	1
#51	M	55	TaHG	high	0
#52	M	66	T2	MIBC	1
#53	M	78	T1HG	high	1
#54	M	73	TaHG	high	1
#55	M	79	T2HG	high	0
#56	M	75	TaHG	high	1
#57	M	72	T1HG	high	1
#58	M	70	T1HG + Cis	high	1
#59	M	70	T2	MIBC	1
#60	M	68	T2	MIBC	1
#61	M	64	T1HG	high	0
#62	M	72	TaHG	high	1
#63	M	72	T1HG	high	1

Cis = carcinoma in situ, TaHG = stage Ta high-grade, MIBC = Muscle Invasive Bladder Cancer. Smoker 0 = No history of cigarette smoking; Smoker 1 = currently smoking or previous history of cigarette smoking.

Table S3. Features of control group. Median age of healthy volunteers: 65 (Q1 = 56.5; Q3 = 69.5). Smoker 0 = No history of cigarette smoking; Smoker 1 = currently smoking or previous history of cigarette smoking.

ID	Gender	Age	Diagnosis	Risk	Smoker (1 = yes)
C1	F	66	negative	/	1
C2	M	83	hyperplasia	/	1
C3	M	24	papilloma	/	0
C4	M	85	prostate disease	/	0
C5	F	43	papilloma	/	1
C6	M	51	papilloma	/	0
C7	M	57	hyperplasia	/	1
C8	F	88	displasia	/	0
C9	F	67	negative	/	0
C10	M	49	negative	/	0
C11	F	69	negative	/	0
C12	M	69	negative	/	0
C13	F	57	negative	/	0
C14	F	59	negative	/	0
C15	M	64	negative	/	0
C16	F	66	negative	/	0
C17	M	65	negative	/	0
C18	M	56	negative	/	0
C19	F	72	negative	/	0
C20	M	64	negative	/	1
C21	M	70	negative	/	1
C22	F	58	negative	/	0
C23	M	54	negative	/	1
C24	F	67	negative	/	0
C25	M	71	negative	/	0
C26	M	67	negative	/	0
C27	M	74	negative	/	0
C28	M	69	negative	/	0
C29	M	54	negative	/	0
C30	F	63	negative	/	0
C31	M	63	negative	/	0
C32	M	64	negative	/	0
C33	M	69	negative	/	1
C34	M	55	negative	/	0
C35	M	85	negative	/	0
C36	M	70	negative	/	0
C37	M	48	negative	/	0

Table S4. Mean Ct and standard deviation (SD) of urine cfmiRNAs analyzed in the total cohort of patients and controls. To overcome the bias linked to data normalization using an external calibrator RNA, we tested the variability of endogenous control miRNAs. Table S4 shows the mean of the Ct and the standard deviation (SD) calculated for the indicated miRNAs in all samples (controls and patients).

miRNA	Mean Ct	SD
miR-125b	28.66	1.25
miR-99a	28.26	1.32
miR-100	28.20	1.35
miR-532	29.67	1.42
miR-29a	25.53	1.42
miR-200a	26.31	1.74
miR-22	29.89	1.82
miR-141	26.91	1.82
miR-193a	31.48	2.03
miR-21	24.76	2.04
miR-200c	25.16	2.17
miR-34a	29.79	2.28
miR-205	28.59	2.69
miR-375	29.75	2.71

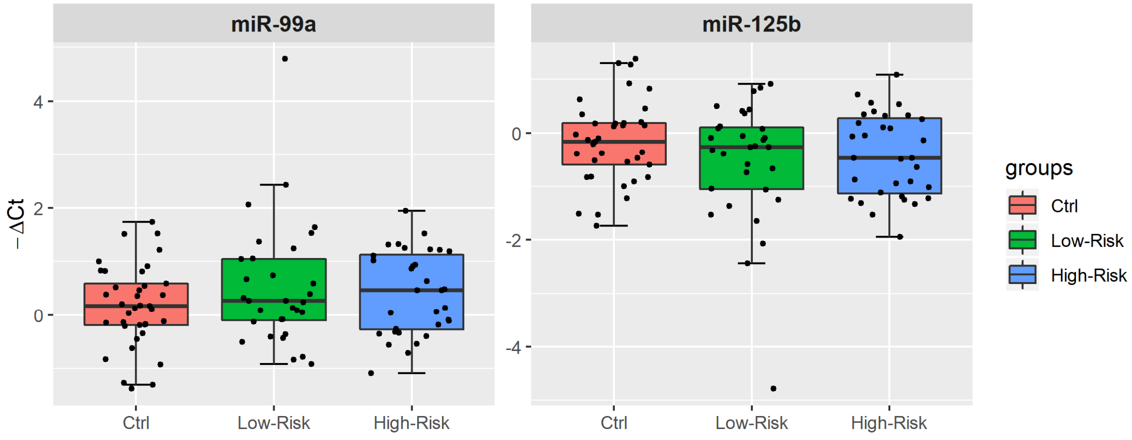

Figure S1. Boxplots with the distribution of the two best candidate normalizers in urine. Urine normalizer miRNA levels in controls (red boxes), low-risk patients (green boxes) and high-risk patients (blue boxes). The y-axis represents the $-\Delta Ct$ of the two candidate normalizers calculated respectively as $-\Delta Ct_{miR-99a} = -(Ct_{miR-99a} - Ct_{miR-125b})$ and $-\Delta Ct_{miR-125b} = -(Ct_{miR-125b} - Ct_{miR-99a})$. miR-125b and miR-99a yielded the lowest SD.

(**A**)

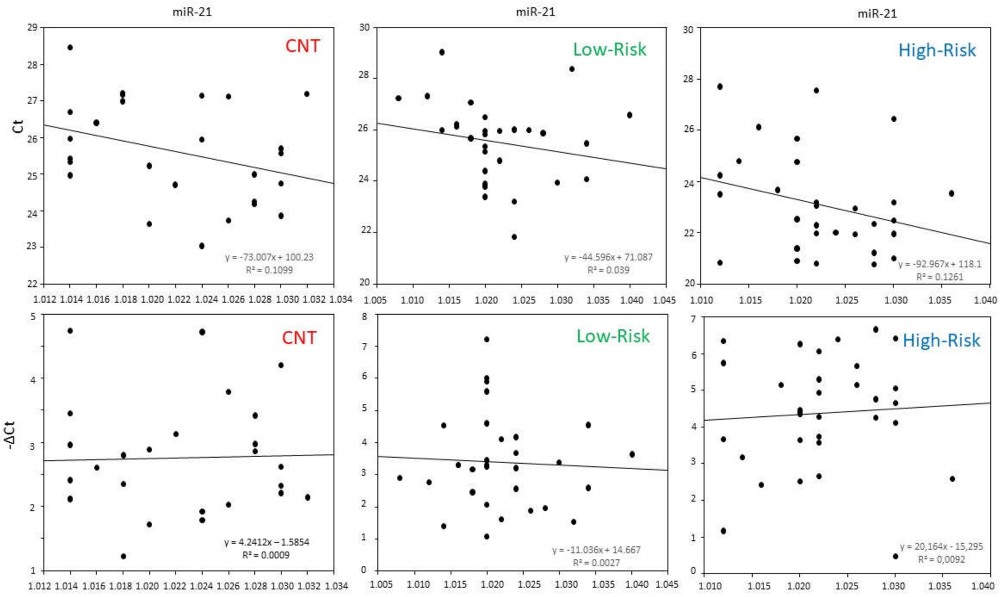
miR-21
CNT
Low-Risk
High-Risk
Ct
-ΔCt
y = -73.007x + 100.23
R² = 0.1099
y = -44.596x + 71.087
R² = 0.039
y = -92.967x + 118.1
R² = 0.1261
y = 4.2412x – 1.5854
R² = 0.0009
y = -11.036x + 14.667
R² = 0.0027
y = 20,164x - 15,295
R² = 0,0092

(B)

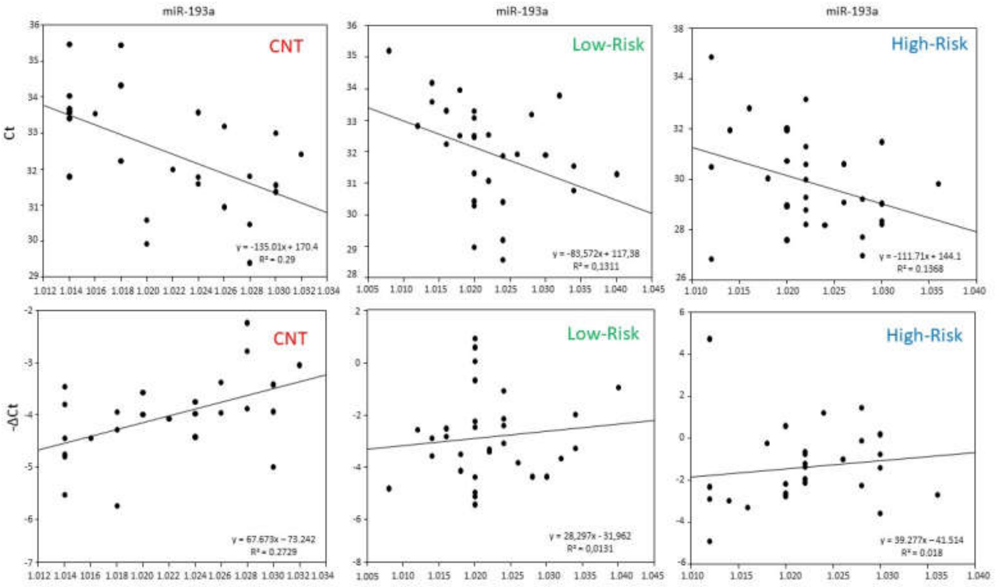
miR-193a
CNT
Low-Risk
High-Risk
Ct
-ΔCt
y = -135.01x + 170.4
R² = 0.29
y = -83,572x + 117,38
R² = 0,1311
y = -111.71x + 144.1
R² = 0.1368
y = 67.673x – 73.242
R² = 0.2729
y = 28,297x - 31,962
R² = 0,0131
y = 39.277x - 41.514
R² = 0.018

(C)

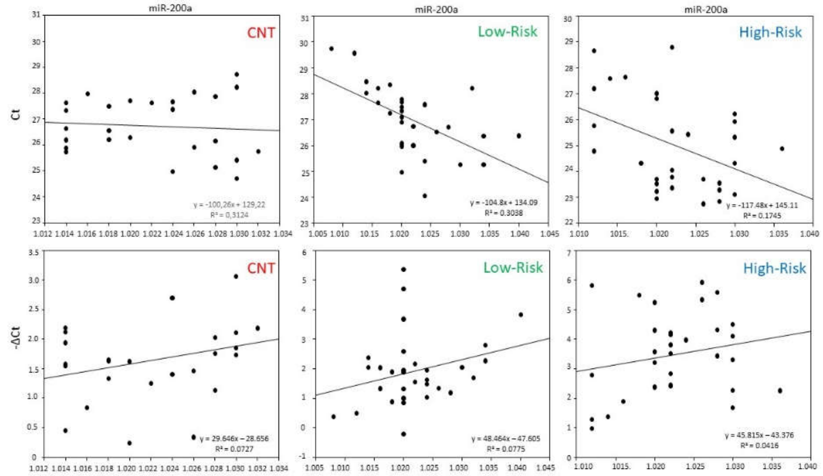

(D)

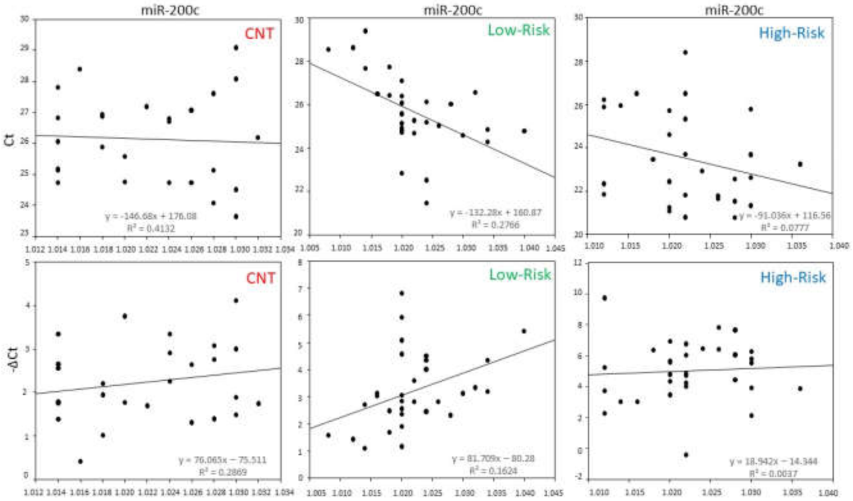

Figure S2. Evaluation of urine specific gravity and miRNA detection. Graphs show correlation analysis between miRNA Ct or −ΔCt using miR-125b (y axis) and urine samples' specific gravity (x axis) in controls, low-risk and high-risk patients (r and *p*-values are indicated). The graphs show that miRNA levels did not correlate with SG if the miRNA was normalized for miR-125b (−ΔCt). (**A**) miR-21 (**B**) miR-193a (**C**) miR-200a. (**D**) miR-200c

(A)

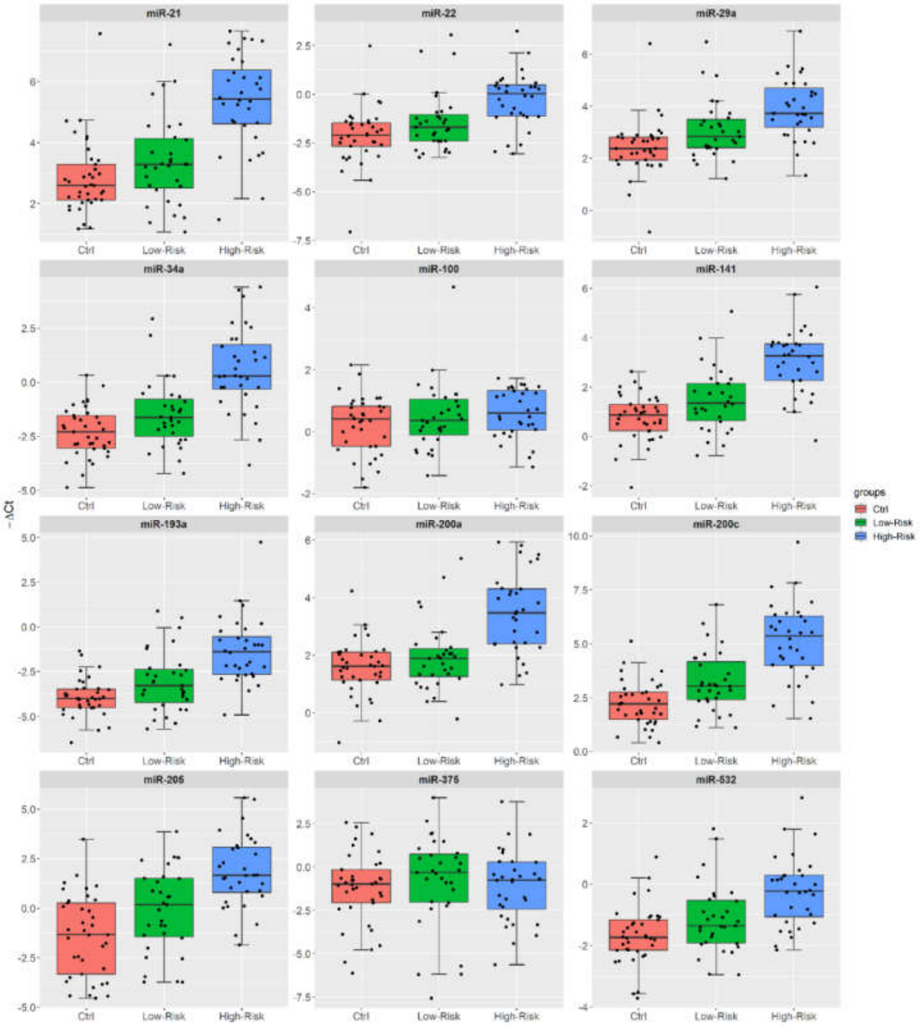
miR-21
miR-22
miR-29a
miR-34a
miR-100
miR-141
miR-193a
miR-200a
miR-200c
miR-205
miR-375
miR-532
−ΔCt
Ctrl
Low-Risk
High-Risk
groups

(B)

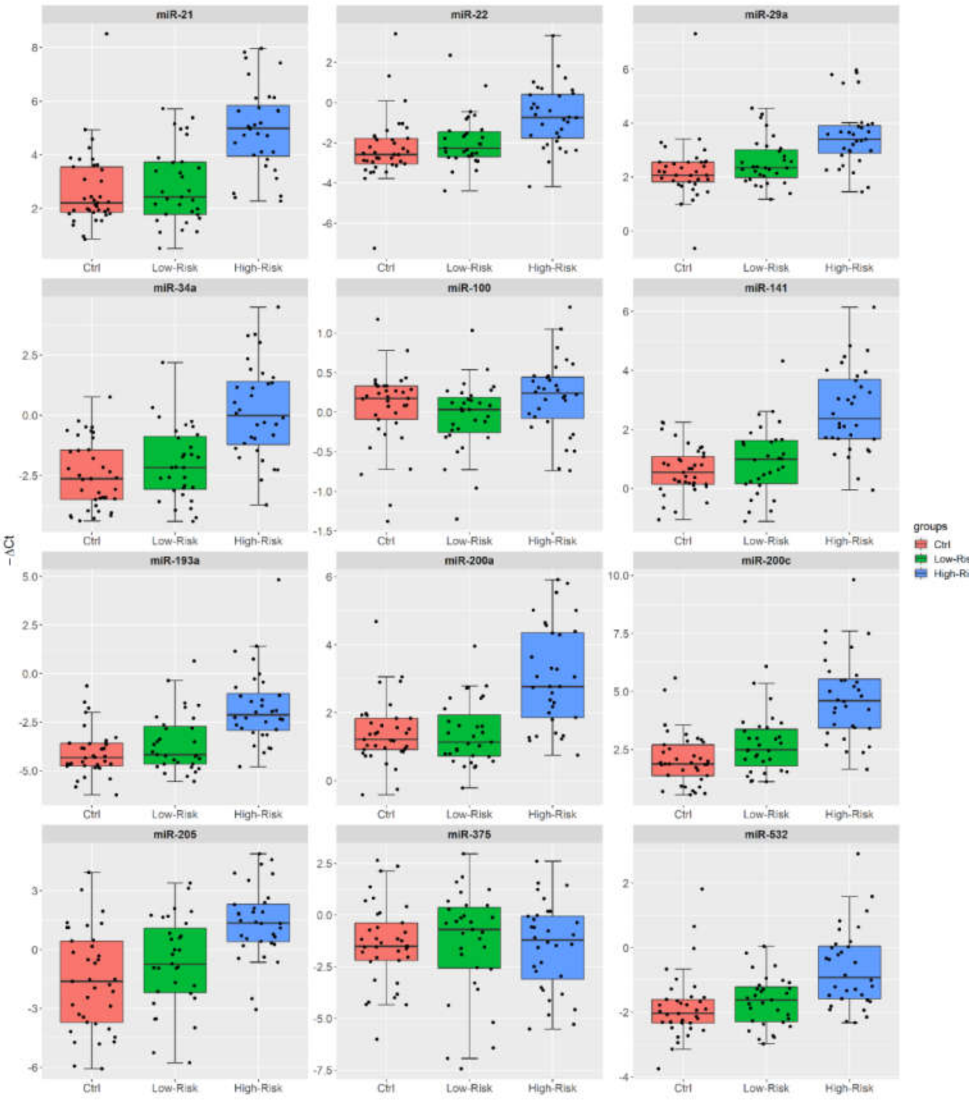

Figure S3. Distribution of urine miRNA levels in controls and BCa patients. (**A**) Boxplots showing the $-\Delta Ct = -(Ct_{miR} - Ct_{miR-125b})$ distribution of urine cfmiRNAs in controls (red boxes), low-risk patients (green boxes) and high-risk patients (blue boxes). (**B**) Boxplots showing the $-\Delta Ct = -(Ct_{miR} - Ct_{miR-99a})$ distribution of urine cfmiRNAs in controls (red boxes), low-risk patients (green boxes) and high-risk patients (blue boxes).

Table S5. Evaluation of urine cfmiRNAs as biomarkers of low-risk *vs* high-risk BCa. The table reports the analyzed miRNAs, the mean Ct measured for low-risk patients (LR) and high-risk (HR) patients. Columns 4, 5 and 6 show, respectively, the fold change (FC), the power and the Bonferroni adjusted p-value (*p-Bonf*) in the comparison of HR *vs*. LR patients by a two-tailed Wilcoxon rank sum test (see Materials and Methods). A univariate logistic regression model was developed for each miRNA, and the significance and the corresponding areas under the ROC curves (AUC) were calculated and reported in the table (last 2 columns).

miRNA	Mean Ct (LR)	Mean Ct (HR)	FC (HR vs. LR)	Power	p-Bonf	Significance in univariate logistic regression	AUC
miR-21	25.54	23.17	3.96	0.9997	5.52E-05	0.0001	0.838
miR-22	30.33	28.81	2.18	0.9449	7.26E-03	0.0070	0.754
miR-29a	25.84	24.60	1.78	0.8927	0.0222	0.0097	0.728
miR-34a	30.41	27.94	4.22	0.9958	1.16E-04	0.0004	0.813
miR-100	28.45	27.93	1.09	0.2037	1	0.5871	0.584
miR-141	27.48	25.48	2.97	0.9974	3.29E-04	0.0003	0.812
miR-193a	31.98	29.87	3.28	0.9790	2.33E-03	0.0013	0.775
miR-200a	27.01	25.08	2.91	0.9975	2.89E-04	0.0003	0.811
miR-200c	25.70	23.40	3.74	0.9929	6.12E-04	0.0003	0.799
miR-205	29.06	26.71	3.88	0.9569	8.04E-03	0.0013	0.752
miR-375	29.75	29.64	-1.22	0.1741	1	0.6337	0.575
miR-532	30.07	28.77	1.87	0.9051	0.0204	0.0055	0.732

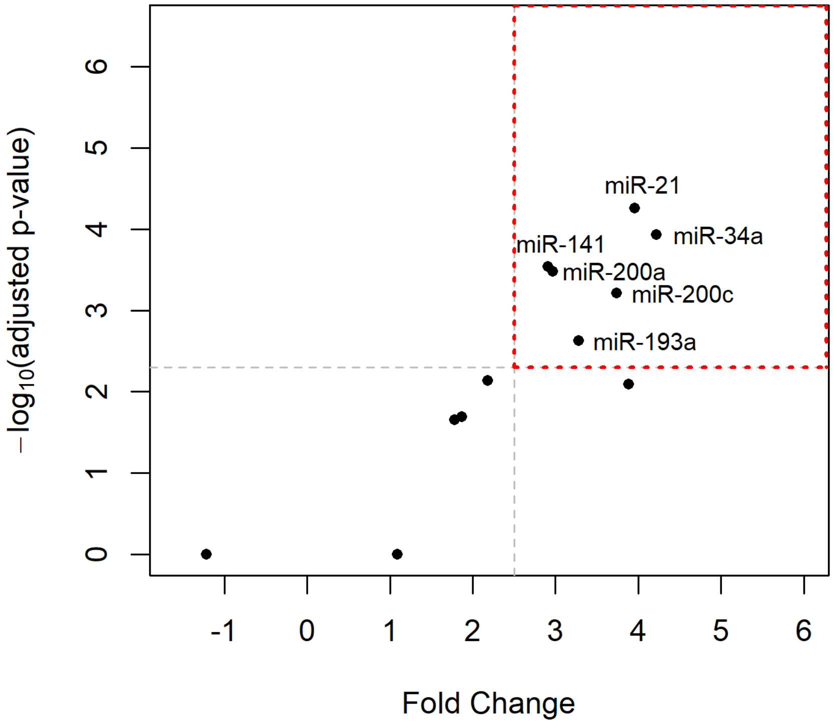

Figure S4. Scatter plot showing the criteria used to identify the six most upregulated urine miRNAs in high-risk vs low-risk patients. The x-axis represents the fold change (FC) obtained by comparing high-risk *vs*. low-risk patients and the y-axis represents the $-\log_{10}$(Bonferroni adjusted p-value). Each miRNA is represented by a dot. The dashed grey lines, corresponding to a Bonferroni-adjusted p-

value of 0.005 (horizontal line) and a FC of 2.5 (vertical line), identify the 6 most significantly up-regulated miRNAs in the upper right quadrant.

Table S6. Univariate logistic regression models for the six miRNAs selected as urine biomarkers of high-risk BCa. The estimated coefficients with the corresponding standard errors (SE), the significance expressed as p-value (P), and odds ratios (OR) along with their 95% confidence intervals (CI) are reported for the six univariate logistic regression models.

miRNA	Coefficient	SE	P	OR	95% CI
miR-21	0.833	0.215	0.0001	2.301	1.570 - 3.694
miR-34a	0.711	0.200	0.0004	2.036	1.434 - 3.171
miR-141	0.931	0.257	0.0003	2.538	1.608 - 4.460
miR-193a	0.608	0.189	0.0013	1.836	1.309 - 2.773
miR-200a	0.908	0.253	0.0003	2.480	1.585 - 4.340
miR-200c	0.756	0.207	0.0003	2.129	1.473 - 3.341

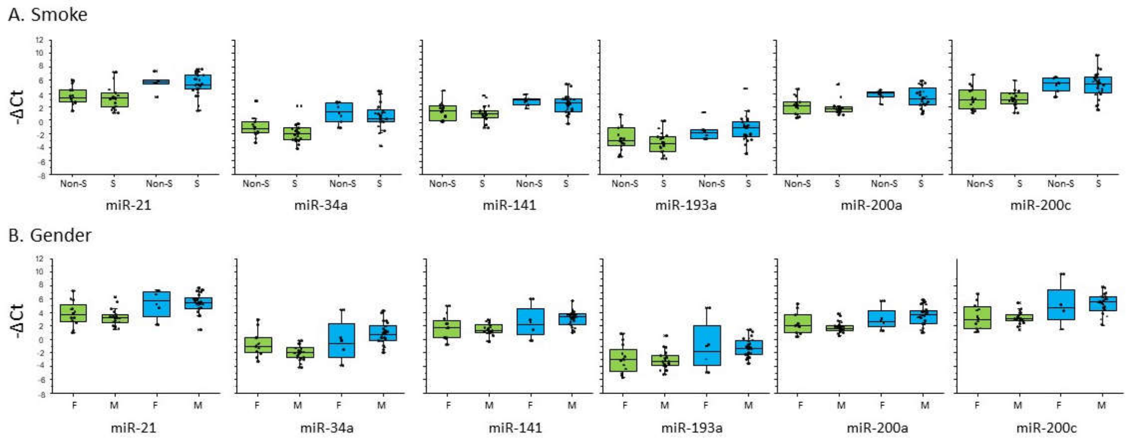

Figure S5. Evaluation of confounding factors: smoking and gender. A comparison of urine miRNA levels in non-smokers (Non-S) vs. smokers (S) and in females (F) vs. males (M) in low-risk and high-risk patients (green boxes and blue boxes, respectively). Low-risk patients: 11 non-smokers, 20 smokers; 12 females and 19 males. High-risk patients: 5 non-smokers, 27 smokers; 4 females and 28 males. Non-S: No history of cigarette smoking; S: currently smoking or previous history of cigarette smoking.

Table S7. Ratios generated by combinations of the 14 urine cfmiRNAs examined. The miRNA ratio approach was applied and 91 ratios were generated. The table shows only the miRNA ratios that were significantly different (two-tailed Wilcoxon rank sum test) between high-risk (HR) and low-risk (LR) patients with Bonferroni adjusted p-value (*p-Bonf*) < 0.05. Seven ratios (highlighted in yellow) were strongly significant with *p-Bonf* < 0.001. Results of this unsupervised approach confirmed the same panel of six miRNAs identified by using miR-125b as normalizer.

miRNA ratio	LR mean	HR mean	p-value (HR vs. LR)	p-Bonf (HR vs. LR)
miR-21/miR-99a	7.25	31.15	1.46E-06	0.00013
miR-141/miR-99a	1.93	6.24	2.78E-06	0.00025
miR-21/miR-125b	10.48	41.49	4.25E-06	0.00039
miR-200c/miR-99a	6.52	26.47	6.93E-06	0.00063
miR-200a/miR-99a	2.62	8.29	7.39E-06	0.00067
miR-21/miR-100	7.52	27.22	8.29E-06	0.00075
miR-34a/miR-125b	0.36	1.51	8.94E-06	0.00081

miR-34a/miR-99a	0.25	1.14	0.00001	0.00127
miR-200a/miR-125b	3.79	11.04	0.00002	0.00202
miR-34a/miR-100	0.26	0.99	0.00002	0.00209
miR-21/miR-29a	1.23	2.86	0.00002	0.00212
miR-141/miR-125b	2.80	8.31	0.00003	0.00231
miR-193a/miR-99a	0.08	0.30	0.00003	0.00316
miR-200c/miR-125b	9.43	35.25	0.00005	0.00429
miR-29a/miR-200c	0.90	0.41	0.00006	0.00568
miR-200a/miR-100	2.72	7.24	0.00007	0.00627
miR-200c/miR-100	6.77	23.13	0.00008	0.00703
miR-141/miR-100	1.99	5.45	0.00008	0.00751
miR-193a/miR-125b	0.12	0.40	0.00018	0.01632
miR-29a/miR-34a	23.72	9.63	0.00018	0.01642
miR-193a/miR-100	0.09	0.26	0.00028	0.02584
miR-22/miR-99a	0.26	0.62	0.00038	0.03423

Table S8. Mean Ct and standard deviation (SD) calculated for miRNAs in plasma samples**.** The analysis was performed on plasma samples from controls C1, C4, C6, C7, C8, C9, C16, C20, C21, C22, C23, C36, C37 and on all BCa patients with the exception of #1 (low-risk), #42 and #52 (MIBC).

miRNA	Mean Ct	SD
miR-21	25.1842	0.7626
miR-29a	27.4301	1.2063
miR-34a	32.9278	1.2117
miR-99a	33.1415	0.9179
miR-100	33.0431	0.9017
miR-125b	31.4583	1.2682
miR-141	35.1484	1.301
miR-193a	32.412	1.0068
miR-200a	34.9919	0.8956
miR-200c	34.8047	1.0191
miR-205	35.5931	1.5877

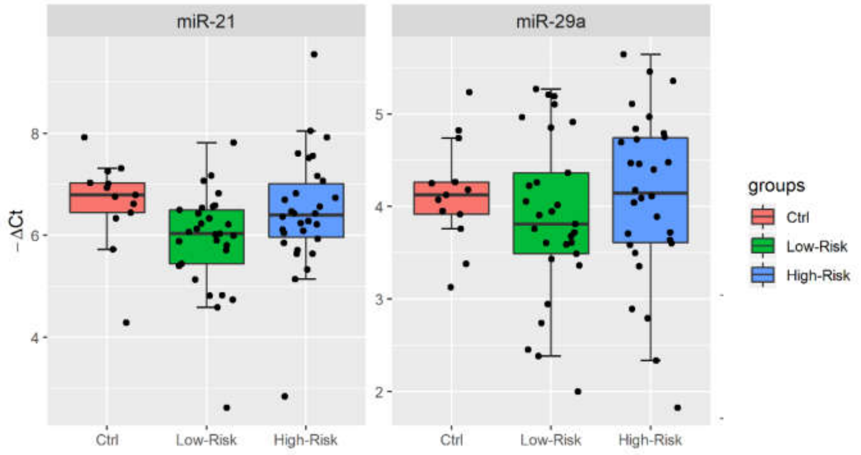

Figure S6. Plasma miRNA levels across groups. The data are from controls and patients indicated in the legend to Table S8. Boxplots show the $-\Delta Ct$ values of the 2 plasma cfmiRNAs with mean Ct <30 normalized for miR-125b. Controls (red boxes), low-risk patients (green boxes) and high-risk patients (blue boxes).

Table S9. Univariate analysis of urine miRNAs associated with EFS in bladder cancer patients. HR: hazard ratio, CI: confidence interval. BH: Benjamini-Hochberg adjusted p-values.

miRNA	Cut-off values		events	Median (95%CI)	Log-rank *p*-value	Log-rank *p*-value [BH]	HR (95%CI)	HR *p*-value
miR-21	1.88	high	18/57	26.8 (19;-)	*0.036*	*0.0599*	-	
		low	1/6	33 (-;-)				
miR-22	-0.095	high	10/22	19.6 (13.2;-)	*0.0243*	*0.0521*	3.0 (1.1-8.3)	*0.0323*
		low	9/41	28.3 (26.8;-)				
miR-29a	4.87	high	5/9	14.1 (4.1;-)	*0.0148*	*0.0477*	3.5 (1.2-10.2)	*0.0225*
		low	14/52	28.3 (21.0;-)				
miR-34a	0.625	high	9/16	19 (8.9;-)	*0.0116*	*0.0477*	3.3 (1.2-8.9)	*0.0177*
		low	10/47	28.3 (26.8;-)				
miR-141	3.135	high	9/19	19.6 (13.2;-)	*0.0642*	*0.0875*	2.5 (0.9-6.6)	*0.0747*
		low	10/43	28.3 (21.0;-)				
miR-193a	-0.63	high	5/11	14.1 (3.9;-)	*0.0092*	*0.0477*	3.8 (1.3-11.0)	*0.0154*
		low	14/52	26.8 (21.0;-)				
miR-200a	3.435	high	10/20	19.6 (13.2;-)	*0.03*	*0.0563*	2.9 (1.0-8.0)	*0.0392*
		low	9/43	28.3 (26.8;-)				
miR-200c	5.66	high	8/14	19.6 (5.5;-)	*0.0078*	*0.0477*	3.5 (1.3-9.3)	*0.0128*
		low	11/49	28.3 (21.0;-)				
miR-205	2.105	high	9/18	19.6 (9.0;-)	*0.0191*	*0.0477*	3.1 (1.1-8.2)	*0.0265*
		low	10/45	28.3 (21.0;-)				
miR-532	0.14	high	7/15	14.1 (5.5;-)	*0.0175*	*0.0477*	3.2 (1.2-8.6)	*0.0239*
		low	12/48	28.3 (21.0)				

Table S10. Univariate analysis of clinical characteristics of bladder cancer patients associated with EFS. HR: hazard ratio, CI: confidence interval. BH: Benjamini-Hochberg adjusted *p*-values.

		Events	Median (95%CI)	Log-rank *p*-value	Log-rank *p*-value [BH]	HR (95%CI)	HR p-value
Risk group	High+MIBC	11/32	23.2 (19.0;-)	0.5673	0.6078	1.3 (0.5-3.4)	0.5698
	Low-Intermediate	8/31	26.8 (13.2;-)			1	
Smoking	Ex	13/32	23.2 (19.0;-)	0.4080	0.4708	1	
	No	3/16	-			0.9 (0.2–3.2)	0.8359
	Yes	3/15	33.0 (14.1;-)			0.4 (0.1–1.7)	0.1964
Intracavitary therapy	No	10/38	26.8 (19.6;-)	0.3876	0.4708	1	
	Yes	9/25	23.2 (13.2;-)			1.5 (0.6–4.0)	0.3912
Age	<75 yrs	13/44	26.8 (21.0;-)	0.0454	0.0681	1	
	>75 yrs	6/17	19.6 (8.9;-)			2.7 (1.0–7.5)	0.0538
Gender	Female	6/16	28.3 (10.1;-)	0.8927	0.8927	1	
	Male	13/47	26.8 (19.6;-)			1.1 (0.3–3.4)	0.8899

Table S11. Urine parameters in patients and controls. * indicates the low/intermediate risk patients, and ** indicates the high-risk/MIBC patients. The "SG" columns report Specific Gravity values measured with Multistix and with a refractometer. Indicated in the "Haemolysis" column is the absence (Negative) or the presence of non-haemolyzed RBC (N-H RBC, score 10–80) or haemolyzed RBC (H RBC, score 25–300). Indicated in the "Protein" column is absence or presence of traces or mg/dL of proteins. In the "LEU" column is indicated the presence or the absence of leukocytes. All the samples were negative for glucose, ketones, bilirubin, urobilinogen and nitrite. ND: Not determined. Data were not available for patients 1,2,4,5 (low/intermediate risk group), patients 34, 35, 36, 37, 62 (high-risk/MIBC group) and for control C2.

Patients	SG with Multistix	SG with refractometry	Haemolysis	pH	PRO	URO	LEU
#6 *	1.000	1.008	H RBC 10	6.5	negative	negative	negative
#7 *	1.030	1.020	Non-H RBC10	6	negative	negative	negative
#8 *	1.000	1.032	Negative	6.5	negative	negative	negative
#9 *	1.015	1.020	H RBC 200	6.5	negative	negative	negative
#38 **	1.015	1.012	H RBC 300	6.5	trace	negative	large
#10 *	1.030	1.024	Negative	6	trace	negative	negative
#39 **	1.031	1.020	Non-H RBC 10	5	30	negative	negative
#40 **	1.030	1.020	H RBC 300	6.5	trace	negative	negative
#41 **	1.030	1.012	H RBC 200	5	negative	negative	negative
#42 **	1.020	1.026	H RBC 300	7.5	negative	negative	negative
#43 **	1.025	1.026	Negative	6	negative	negative	negative
#44 **	1.030	1.030	H RBC 300	6	trace	negative	negative
#11 *	1.030	1.020	H RBC 300	6	negative	negative	negative
#12 *	1.030	1.020	Negative	6	negative	negative	negative
#45 **	1.030	1.020	H RBC 300	6.5	trace	negative	negative
#46 **	1.030	1.028	H RBC 300	6.5	negative	negative	negative
#13 *	1.025	1.020	Negative	6.5	negative	negative	negative
#47 **	1030	1018	Non-H RBC 10	6.5	negative	negative	negative
#14 *	1.030	1.022	Negative	6	negative	negative	negative
#48 **	1.030	1.030	H RBC 300	6.5	negative	negative	negative
#15 *	1.025	1.020	Non-H RBC 10	6	negative	negative	negative
#16 *	1.030	1.040	Non-H RBC 10	5	negative	negative	negative
#49 **	1.030	1.030	H RBC 300	6	negative	negative	negative
#17 *	1.005	1.020	Negative	6.5	negative	negative	negative
#18 *	1.005	1.018	Negative	6.5	negative	negative	negative
#19 **	1.030	1.022	Non-H RBC 10	5	negative	negative	negative
#50 **	1.030	1.022	H RBC 25	6	trace	negative	negative
#51 **	1.010	1.024	H RBC 300	7	30	negative	negative
#20 *	1.030	1.020	H RBC 300	6	negative	negative	negative
#52 *	1.015	1.036	Non-H RBC 80	6.5	100	negative	negative
#21 *	1.030	1.034	Non-H RBC 80	6	100	negative	negative
#53 **	1.020	1.012	H RBC 300	6	negative	negative	negative
#22 *	1.030	1.024	Negative	6	negative	negative	negative
#54 **	1.025	1.020	H RBC 300	6.5	30	negative	negative
#55 **	1.020	1.020	H RBC 300	7	ND	negative	negative
#56 **	1.005	1.014	RBC 300	8.5	negative	negative	negative
#23 *	1.025	1.034	Non-H RBC 10	7.5	negative	negative	negative
#57 **	1.010	1.022	Negative	7/7.5	trace	negative	negative
#58 **	1.025	1.020	H RBC 300	6.5	30	negative	negative
#59 **	1.030	1.030	H RBC 300	6.5	300	negative	negative
#60 **	1.030	1.028	H RBC 300	6	100	negative	negative
#24 *	1.020	1.014	Negative	6	negative	negative	negative
#25 *	1.030	1.028	H RBC 200	6	negative	negative	negative
#61 **	1.030	1.028	H 300	6	100	negative	negative
#26 *	1.025	1.020	Negative	6	negative	negative	negative
#27 *	1.015	1.024	Negative	6	negative	negative	ND
#31 *	1.015	1.020	Negative	7	30	negative	negative
#28 *	1.020	1.018	H RBC 200	6	negative	negative	negative
#29 *	1.015	1.016	Non H-RBC 80	7	300	negative	negative
#30 *	1.010	1.012	ND	6	30	negative	ND
#63 **	1.020	1.030	Negative	6.5	30	negative	negative
#32 *	1.015	1.024	H RBC 200	6	negative	negative	ND
#33 *	1.030	1.026	H RBC 300	6	ND	negative	negative

Healthy controls	SG with Multistix	SG with refractometry	Haemolysis	pH	PRO	LEU
**C10**	ND	1.014	ND	ND	ND	ND
**C11**	1.010	1.014	Negative	6.5	negative	negative
**C12**	1.020	1.014	Negative	6	negative	negative
**C13**	1.025	1.020	Negative	6.5	negative	trace
**C14**	1.030	1.028	Negative	6.5	negative	negative
**C15**	1.010	1.018	Negative	6.5	negative	negative
**C16**	1.030	1.036	Negative	6.5	negative	negative
**C17**	1.025	1.024	Negative	6.5	negative	negative
**C18**	1.000	ND	Negative	8	negative	negative
**C19**	1.010	1.014	Negative	6.5	negative	negative
**C20**	1.030	1.028	Negative	6	negative	negative
**C21**	1.025	1.028	Negative	6	negative	negative
**C22**	1.015	1.016	Negative	6	negative	negative
**C23**	1.030	1.028	Negative	6	negative	negative
**C24**	1.015	1.014	Negative	6	negative	negative
**C25**	1.030	1.030	Negative	6	negative	negative
**C26**	1.005	1.018	Negative	6	negative	negative
**C27**	1.015	1.018	Negative	6.5	negative	negative
**C28**	1.025	1.022	Negative	5	negative	negative
**C29**	1.020	1.030	Negative	5	negative	negative
**C30**	1.015	1.014	Negative	5	negative	negative
**C31**	1.015	1.026	Negative	5	negative	negative
**C32**	1.030	1.030	Negative	5	negative	negative
**C33**	1.025	1.026	Non-H RBC 80	6	negative	negative
**C35**	1.005	1.014	Negative	7	negative	negative
**C36**	1.000	ND	Non- H RBC 80	8.5	negative	negative
**C34**	1.020	ND	Negative	6.5	negative	negative
**C37**	1.010	ND	Non-H 80	7.5	ND	negative
**C1**	1.030	1.014	H RBC 200	6.5	0.3	trace
**C6**	ND	ND	Negative	6.5	negative	negative
**C7**	1.030	1.024	Negative	6	trace	negative
**C8**	1.005	ND	H RBC 300	7.5	negative	negative
**C9**	1.010	1.020	Negative	7.5	negative	negative
**C5**	1.000	ND	Negative	7.5	negative	negative
**C3**	1.015	ND	Negative	6.5	negative	negative
**C4**	1.030	1.030	Negative	5	negative	negative
